# Scalable Synthesis and Electrochemical Properties of Porous Si-CoSi_2_-C Composites as an Anode for Li-ion Batteries

**DOI:** 10.3390/ma14185397

**Published:** 2021-09-18

**Authors:** Hyungeun Seo, Hae-Ri Yang, Youngmo Yang, Kyungbae Kim, Sung Hyon Kim, Hyunseung Lee, Jae-Hun Kim

**Affiliations:** 1 School of Materials Science and Engineering, Kookmin University, Seoul 02707, Korea; 20131101@kookmin.ac.kr (H.S.); didgofl8282@kookmin.ac.kr (H.-R.Y.); yym0116@kookmin.ac.kr (Y.Y.); kyungbaekim@kookmin.ac.kr (K.K.); 2 Department of Fashion Design, Kookmin University, Seoul 02707, Korea; kim_sunghyon@kookmin.ac.kr; 3 Department of Fashion Industry, Incheon National University, Incheon 22012, Korea; srwalpha@inu.ac.kr

**Keywords:** Si-based materials, cobalt silicide, porous structure, composite, anode, Li-ion battery

## Abstract

Si-based anodes for Li-ion batteries (LIBs) are considered to be an attractive alternative to graphite due to their higher capacity, but they have low electrical conductivity and degrade mechanically during cycling. In the current study, we report on a mass-producible porous Si-CoSi_2_-C composite as a high-capacity anode material for LIBs. The composite was synthesized with two-step milling followed by a simple chemical etching process. The material conversion and porous structure were characterized by X-ray diffraction, X-ray photoelectron spectroscopy, and electron microscopy. The electrochemical test results demonstrated that the Si-CoSi_2_-C composite electrode exhibits greatly improved cycle and rate performance compared with conventional Si-C composite electrodes. These results can be ascribed to the role of CoSi_2_ and inside pores. The CoSi_2_ synthesized in situ during high-energy mechanical milling can be well attached to the Si; its conductive phase can increase electrical connection with the carbon matrix and the Cu current collectors; and it can accommodate Si volume changes during cycling. The proposed synthesis strategy can provide a facile and cost-effective method to produce Si-based materials for commercial LIB anodes.

## 1. Introduction

The recent rapid development of electric vehicles and mobile electronics requires high-power and long-lasting rechargeable batteries. Li-ion batteries (LIBs) are currently considered to be the most attractive energy storage devices because of their reasonable cost and high electrochemical performance. Li metal batteries have been actively investigated as high-energy-density systems, but several issues still hinder their commercialization. In LIBs, graphite is commonly used as an anode material because of its high initial coulombic efficiency, low operating potential, and long cycle life. However, its limited specific capacity, theoretically 372 mAh g^–1^ from intercalation chemistry, is an obstacle to increasing LIB energy density. Therefore, graphite needs to be replaced with high-capacity materials. Li-alloy-based materials have been considered the most attractive candidates due to their electrochemical Li-alloying during cycling [1,2,3,4].

Si is one of the most popular Li-alloy-based materials. It has a high theoretical specific capacity of 3580 mAh g^–1^ in its final Li_15_Si_4_ phase after electrochemical lithiation but exhibits poor cycling performance caused by mechanical pulverization of active materials during Li insertion and extraction processes [1,2,3,4]. To overcome this drawback, many researchers have adopted strategies to buffer the huge volume changes that occur during cycling. For example, downsizing Si particles to nanoscale and incorporating structures like nanospheres, nanowires, and nanotubes can alleviate volume changes [5,6,7,8,9]. Moreover, Si has been prepared in the form of composites and/or compounds with secondary phases of carbon or inactive/less active to Li^+^ ions; these include materials such as Cu, Ni, Fe, SiN, Al_2_O_3_, Nb_2_O_5_, TiO_2_, and SiO_x_ [10,11,12,13,14,15,16,17,18,19,20,21,22].

The electrical conductivity of the active materials is a very important property of LIB anodes. High electrical conductivity can help an electrode obtain high initial coulombic efficiency and good rate performance. However, Si exhibits poor electrical conductivity (2.52 × 10^–4^ S m^–1^) [23], which creates large resistance during Li insertion/extraction cycling. This characteristic can accelerate performance degradation by electrically isolating the Si materials from the current collectors. To guarantee the electrochemical properties of electrodes, we can enhance their electrical conductivity by adding conductive materials, such as metals and carbon [24]. Additionally, a porous structure has been proved to benefit Li-alloy-based electrodes because it can provide extra space for buffering volume changes of active materials [25,26,27,28,29,30,31,32]. A highly conductive metal and/or compound that is incorporated into Si and that maintains a porous structure through in situ synthesis can be a great option for Si composite materials. Among the choices of secondary phases to buffer volume changes and to increase electrical conductivity, Co-based materials can be a good candidate because they show high electrical conductivity, are electrochemically inactive/less active to Li^+^ ions, and can improve the structural stability of Si. This led us to try to identify a cost-effective method of synthesizing porous Si composites that incorporate Co-based material.

In this study, we propose a strategy for producing a porous Si-CoSi_2_-C nanocomposite as an anode for LIBs. The nanocomposite was synthesized through a simple and cost-effective process that uses high-energy mechanical milling (HEMM) and chemical etching. Si, Mg_2_Si, and Co_3_O_4_ powders were selected as starting materials for the formation of Si-CoSi_2_-MgO composites by solid-state reactions during HEMM. We selected Mg_2_Si instead of Mg as a reducing agent for Co_3_O_4_ to provide additional Si for the composite. After a second milling to provide carbon, the resulting MgO was eliminated with chemical etching. A porous Si-CoSi_2_-C composite was successfully synthesized with this process. The material was thoroughly characterized in several material and electrochemical analyses. The composite electrode demonstrated a high reversible capacity of 1193 mAh g^–1^ with a 75.3% initial coulombic efficiency and much improved capacity retention compared with a Si-C electrode.

## 2. Experimental Procedure

Material preparation: Porous Si-CoSi_2_-C composites were prepared via a two-step milling process and chemical etching. In the first step, Si (−60 mesh, Sigma-Aldrich, Seoul, Korea), Mg_2_Si (−20 mesh, Sigma-Aldrich, Seoul, Korea), and Co_3_O_4_ (<10 μm, Sigma-Aldrich, Seoul, Korea) powders in a molar ratio of 6:2:1 with stearic acid (1 wt%) as a process controlling agent were mixed and transferred into a steel vial (65 cm^3^) with stainless steel balls at a ball-to-powder ratio of 30:1. HEMM was carried out under an Ar atmosphere for 12 h. After this milling, the resulting composite was milled with activated carbon (Super P) at a mass ratio of 8:2 for 6 h to incorporate carbon. The resulting material was treated with 2 M HCl solution for 3 h and then washed and dried at 60 ℃ for 6 h.

Material characterization: To analyze the crystal structure of the composite, X-ray diffraction (XRD, Rigaku D/MAX-2500V, Tokyo, Japan) was performed. X-ray photoelectron spectroscopy (XPS, Thermo Scientific, Shanghai, China) was used to examine the changes in chemical and bonding states from pristine material to composite. Raman spectroscopy with Renishaw instruments was used to analyze the composite’s bond structure. The morphology and surface structure of the composite were observed with field-emission scanning electron microscopy (FE-SEM, JEOL JSM-7401F, Tokyo, Japan) and high-resolution transmission electron microscopy (HR-TEM, JEOL ARM-200F, Tokyo, Japan) with energy-dispersive X-ray spectroscopy (EDS). The Brunauer–Emmett–Teller (BET) surface area of the porous composite was measured with a N_2_-physical adsorption instrument (Micromeritics TriStar II 3020, Norcross, UK) at 77K.

Electrochemical measurements: For the electrochemical cell tests, working electrodes were prepared as follows: The active material (70 wt%), a conducting agent (Super P, 15 wt%), and a binder (polyacrylic acid, 15 wt%) were dissolved in deionized water. Then, the slurry was coated onto copper foil current collectors. After coating, the electrodes were pressed and dried under vacuum at 80 °C for 12 h. CR2032 coin-type half-cells were assembled in an Ar-filled glove box. A Li foil piece was used as a counter/reference electrode, and porous polyethylene films were employed as separators. The electrolyte was 1 M LiPF_6_ in ethylene carbonate and diethyl carbonate (3:7 by volume ratio) with an additive of fluoroethylene carbonate (10 wt%). All the cells were galvanostatically tested between 0.001 and 2.0 V (vs. Li^+^/Li) using specific currents of 100–5000 mA g^–1^ at room temperature. Electrochemical impedance spectroscopy (EIS) was performed from 1 MHz to 0.1 Hz at an amplitude of 15 mV.

## 3. Results and Discussion

**Figure 1:**
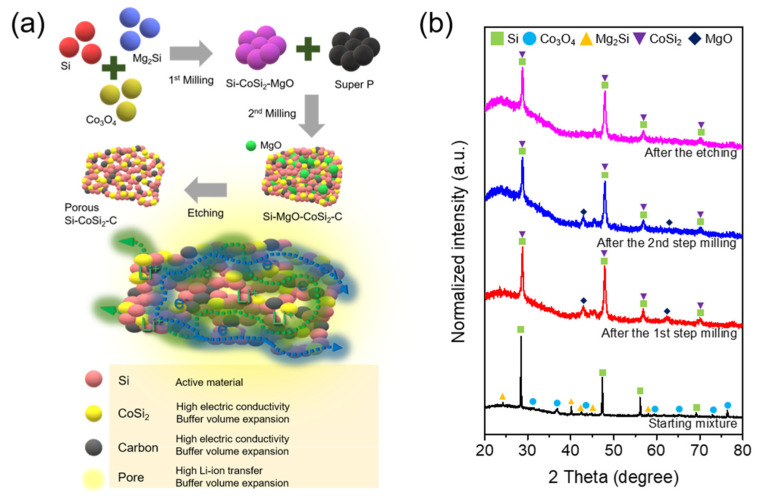
(**a**) Schematic of the porous Si-CoSi_2_-C composite with the roles of each component and (**b**) XRD patterns of the pristine mixture and the samples after each synthesis.

A schematic of the porous composite as an anode material is shown in Figure 1a. The material is composed of Si, CoSi_2_, and carbon with internal pore space. Si is the active material that reversibly stores Li^+^ ions to create a high-capacity anode. The CoSi_2_ that is produced by conversion from Co_3_O_4_ during HEMM is well attached to the Si, increasing electrical conductivity as well as buffering Si volume changes during cycling. The composite’s carbon also buffers volume change and facilitates electron and Li^+^ ion transport as is well reported in the literature [33,34,35]. The inside pores generated by chemical etching aid in absorbing Si volume expansion and allow fast Li^+^ ion transport through the electrolyte. HEMM and etching are viable processes for the mass-production of this elaborate Si composite microstructure. Composite materials and their electrochemical properties were investigated as follows:

Figure 1b shows the XRD patterns of the starting and synthesized materials at each step. The starting materials were Si (ICDD-JCPDS No. 27-1402), Mg_2_Si (ICDD-JCPDS No. 34-0458), and Co_3_O_4_ (ICDD-JCPDS No. 42-1467). After the first milling for 12 h, the diffraction peaks of Mg_2_Si and Co_3_O_4_ disappeared and new diffraction peaks attributable to MgO (ICDD-JCPDS No. 04-0829) were observed. Moreover, the peaks at approximately 28.4°, 47.1°, 56.2°, and 69.2° were broadened and slightly shifted to the right. This can be clearly seen around 28° in the enlarged profiles of Figure S1 and indicates that Si still existed and that a CoSi_2_ phase was created after the HEMM process. The crystal structures of both Si and CoSi_2_ phases are very similar, and, thus, the generation of CoSi_2_ is further confirmed later in this paper. The reaction chemistry can be expressed as follows:

6Si + 2Mg_2_Si + Co_3_O_4_ → 2Si + 4MgO + 3CoSi_2_ (1)

After the second milling to incorporate carbon, the XRD pattern was similar, and the peak intensity slightly decreased because the phases did not change from the amorphous activated carbon addition. The carbon incorporation was confirmed by the presence of D and G bands in the Raman spectrum (Figure S2). After the etching process, the diffraction peaks for the MgO phase disappeared, indicating that MgO was removed to generate pores inside the composite. A peak at approximately 45.5° was observed for all synthesized samples. This can be ascribed to metallic Fe, which could be an impurity produced from the steel balls and vial during milling.

**Figure 2:**
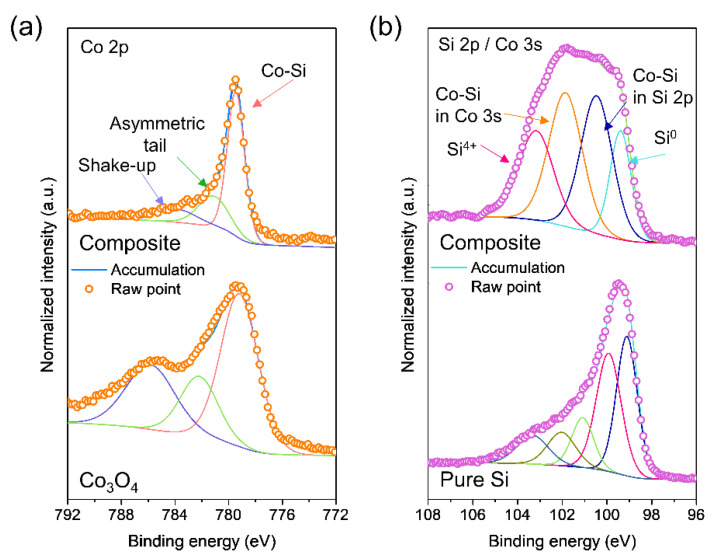
XPS core-level spectra of the composite: (**a**) Co 2p and (**b**) Si 2p/Co 3s.

XPS analysis was performed to confirm the chemical states of each element in the synthesized composites and starting materials. For surface cleaning, Ar sputtering was performed for 300 s. Figure 2a shows the Co 2p core-level spectra with deconvoluted profiles for the materials. The shape of the overall spectrum was changed because Co_3_O_4_ was transformed into CoSi_2_ during HEMM. The Co 2p XPS spectra of the composite were deconvoluted into three sub-profiles. The two profiles at high binding energy can be assigned to a shake-up satellite and asymmetric tail [36,37]. The main peak at approximately 777.5 eV corresponds to the Co-Si bond in CoSi_2_ as reported in the literature [36,37,38,39]. The Si 2p core-level spectra of the materials are shown in Figure 2b. Since these spectra overlap the Co 3s core-level spectra in the same binding energy range, they are plotted in the same graph. The profiles centered at 103.5 and 99.4 eV can be ascribed to Si^4+^ and Si^0^, which originated from the Si particles in the composite. The surface of Si is usually oxidized. The two sub-profiles at the medium range (101.8 and 100.5 eV) can be attributed to the Co-Si bond, which was also detected in the Co 2p spectra. The peak at 101.8 eV might be related to the Co 3s spectrum as reported previously [36,37]. Some Si states (Si^3+^, Si^2+^, and Si^1+^) could overlap in this medium binding energy range. As the Ar ion sputtering time increases, the deconvoluted XPS profiles appear to be similar in both the Co 2p and Si 2p/Co3s spectra (Figure S3). These XPS analyses show that the Si and CoSi_2_ phases were formed during HEMM.

**Figure 3:**
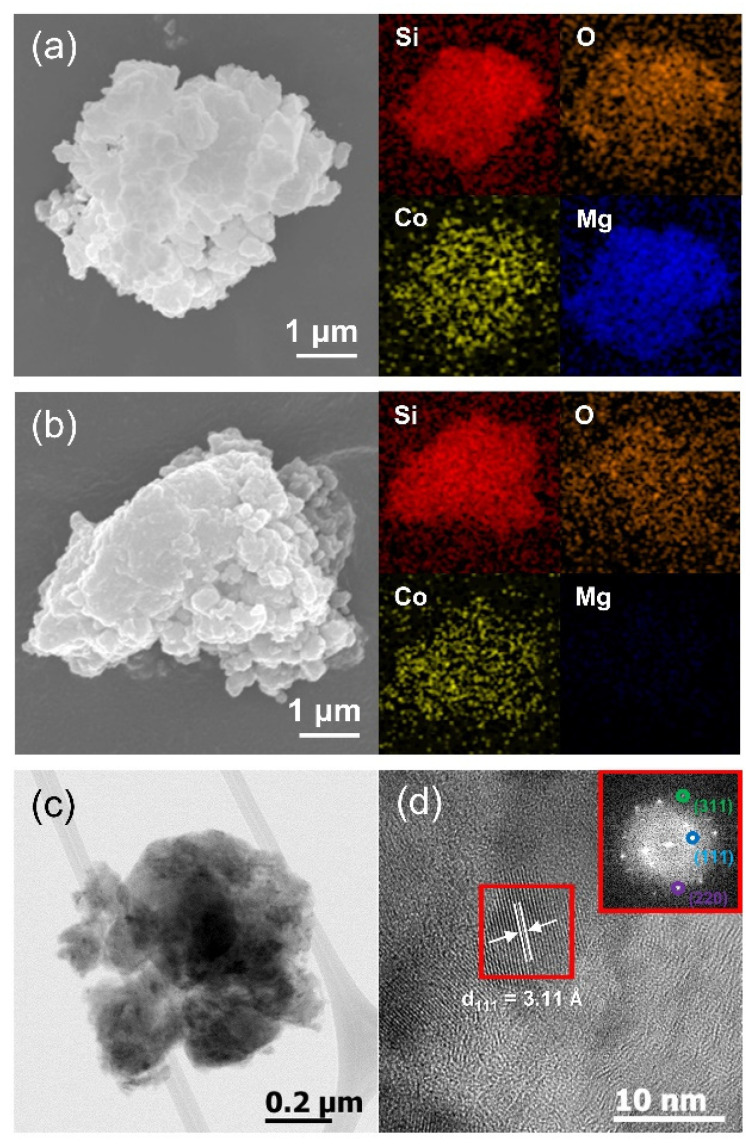
SEM images with EDS elemental mapping results for each element: (**a**) before and (**b**) after the etching process; (**c**) TEM image and (**d**) HR-TEM image (inset: FFT pattern).

Figure 3 shows the electron microscopy results. FE-SEM images with their corresponding EDS elemental mappings of the sample after two-step milling prior to chemical etching are shown in Figure 3a. The size of secondary particles is a few microns; they are composed of primary particles that are tens to hundreds of nanometers in size. The starting materials consisted of micron-sized particles that became nano-sized primary particles during HEMM. It can be seen from the EDS mapping results that the Si, O, Co, and Mg elements were uniformly dispersed in the composite. After etching, the Mg signal disappeared and the signal intensity of O decreased (Figure 3b). This indicates that MgO was removed, forming the porous structure seen in the TEM image of Figure 3c. An HR-TEM image is displayed in Figure 3d. Some crystallites were observed; the measured d-spacing value of 3.11 Å can be attributed to the (111) reflection of Si or CoSi_2_ because the crystal structures of both materials are very similar, as shown in the XRD results. The inset fast Fourier transform (FFT) pattern of the figure also reveals several reflection planes of the two phases.

**Figure 4:**
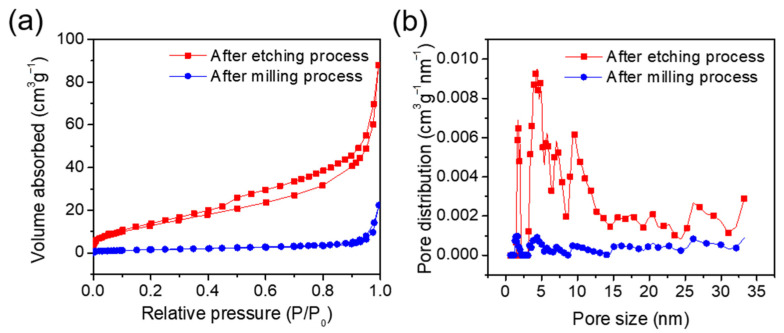
(**a**) N_2_ adsorption and desorption isotherm and (**b**) pore size distribution before and after etching.

The porous structure formation was investigated with a BET surface area measurement on the Si-CoSi_2_-C composite before and after chemical etching. The surface area and pore size distribution were evaluated using nitrogen adsorption and desorption analysis with the results shown in Figure 4. The relative pressure showed a hysteresis loop, which indicates the formation of mesopores (Figure 4a). The BET specific surface area of the milled composite sample at P/P_0_ = 0.99 was 5.64 m^2^ g^–1^. After etching, the porous Si-CoSi_2_-C composite exhibited a surface area of 50.1 m^2^ g^–1^, about ten times higher than before. This means that the etching process for removing MgO was successful and the pores inside the composite were well created. The pore volumes before and after etching were estimated to be 0.034 and 0.130 cm^3^ g^–1^, respectively. The inside pore volumes were greatly increased by the etching process. Figure 4b shows the pore size distribution plot. After etching, it can be seen that the 5 to 15 nm pores were predominantly the ones whose population increased, particularly the micropores of ~5 nm. We expect that the porous structure will help alleviate the volume changes of Si particles and facilitate fast ionic transport via electrolyte penetration.

**Figure 5:**
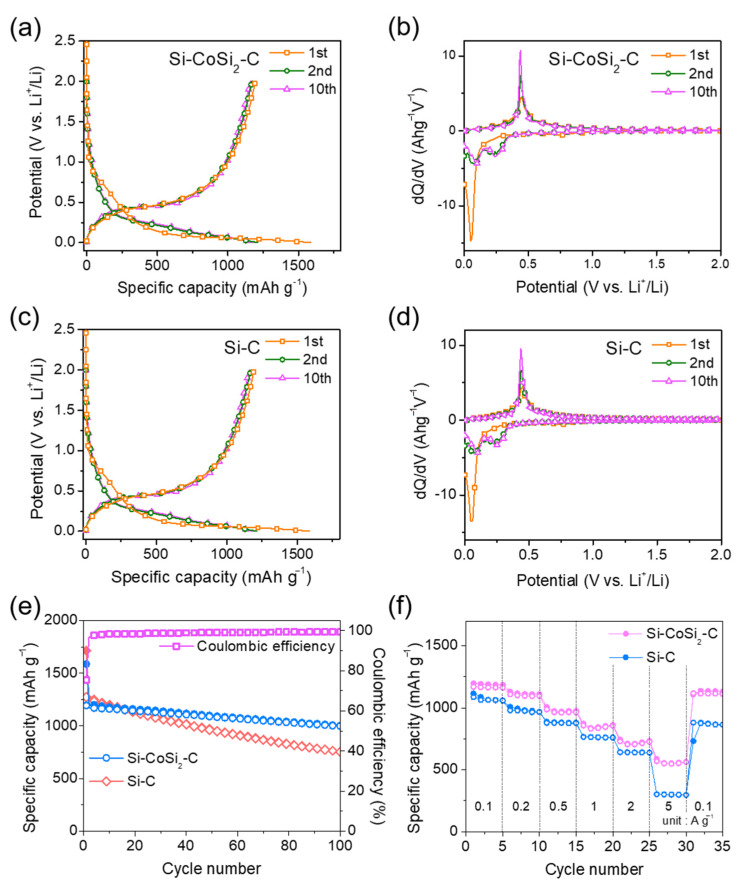
(**a**) Voltage profiles and (**b**) DCPs of the porous Si-CoSi_2_-C composite electrode; (**c**) voltage profiles and (**d**) DCPs of the Si-C composite electrode; (**e**) cycle performance and (**f**) rate performance of both porous Si-CoSi_2_-C and Si-C composite electrodes.

Figure 5 shows the electrochemical properties of the synthesized porous Si-CoSi_2_-C composite electrode for LIBs. The voltage profiles were obtained at a specific current of 100 mA g^–1^ (Figure 5a). The specific discharge and charge capacities for the first cycle were 1586 and 1193 mAh g^–1^, respectively, and the initial coulombic efficiency was about 75.2%. To investigate the electrochemical reaction mechanism of the electrode during Li^+^ insertion and extraction cycling, the voltage profiles for the first, second, and tenth cycles were converted to differential capacity plots (DCPs) with the results shown in Figure 5b. During the first discharge, a small broad peak appeared at around 0.75 V vs. Li^+^/Li (see enlarged profiles in Figure S4); this peak cannot be seen in the second and tenth cycles. This indicates that this reaction was not reversible and could be an irreversible formation of solid electrolyte interphase (SEI) layers on the material surface; this has been well reported in the literature [40,41,42]. A sharp peak was detected below 0.1 V vs. Li^+^/Li in the first discharge corresponding to the electrochemical Li-Si alloying reaction. The final phase of full lithiation is known as the Li_15_Si_4_ phase [43,44]. During the discharge reactions, a sharp peak at approximately 0.45 V vs. Li^+^/Li can be seen in each reaction. This is attributed to the electrochemical Li de-alloying reaction from the lithiated Si in the composite [43,44]. The DCPs exhibited typical electrochemical reactions of Si materials with Li [43,44], revealing that the CoSi_2_ phase was not active to Li^+^ ions. The cyclic voltammograms of the electrode are given in Figure S5, and a similar mechanism can be seen. The reference electrode (Si-C) was prepared from pure Si and activated carbon powders using HEMM to examine the effects of CoSi_2_ and a porous structure. A ratio of Si to carbon (3:7 by weight) was set to a reversible capacity similar to the composite electrode. The voltage profiles and DCPs of the reference electrode are given in Figure 5c,d, respectively. The discharge and charge capacities were 1710 and 1288 mAh g^–1^, respectively, and the initial coulombic efficiency was 75.3%.

Figure 5e shows the cycle performance of the composite and reference electrode measured at a specific current of 100 mA g^–1^. With regard to cycling stability, the synthesized porous composite electrode exhibited much better cycling performance than that of the Si-C electrode. This can be attributed to the effect of CoSi_2_ and internal pores. Both can absorb the volume changes of Si during cycling, and the in situ formed CoSi_2_ phase can provide internal electrical Si contact with better conductivity. The composite electrode exhibited a high reversible capacity of 1005 mAh g^–1^ after 100 cycles. The morphology and microstructure of the Si-CoSi_2_-C composite electrode before and after the long-term cycling are displayed in Figure S6. The FE-SEM images after 100 cycles were similar to those of the pristine electrode, which supported the improved capacity retention property. Figure 5f presents the rate performance of the porous Si-CoSi_2_-C and Si-C composite electrodes at varied specific currents from 100 mA g^–1^ to 5 A g^–1^. To stabilize performance, the electrode was pre-cycled 10 times before the test. Both the discharge and the charge processes were measured at the same current. The composite electrode demonstrated much improved capacity retention at high currents. This enhanced rate performance can be ascribed to the effect of CoSi_2_ with its high electrical conductivity for fast electronic transport and to the porous structure for fast Li^+^ diffusion via facile electrolyte penetration to the inside of the electrode [45,46].

**Figure 6:**
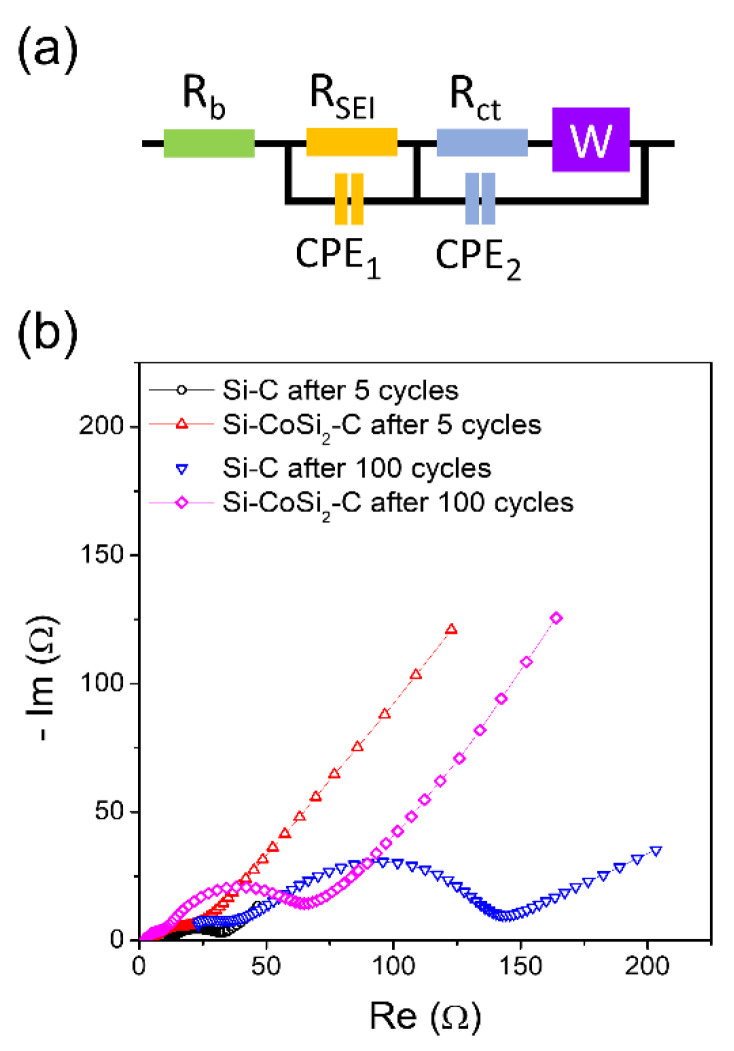
(**a**) Equivalent circuit model and (**b**) Nyquist plots of the Si-CoSi_2_-C and Si-C composite electrodes after 5 and 100 cycles.

To understand the enhanced rate performance of the Si-CoSi_2_-C composite, the interfacial resistance between the electrodes and electrolytes was measured using EIS. Figure 6 shows the equivalent circuit model and Nyquist plots of the Si-CoSi_2_-C and Si-C composite electrodes after 5 cycles and 100 cycles (see enlarged plots in Figure S7). The high-frequency intercept can be generally attributed to the bulk resistance of the electrodes and electrolytes. The interfacial resistance can be determined from the diameter of the first semicircle, which is the intermediate frequency intercept. The straight line in the low-frequency region is related to the Warburg impedance raised by Li-ion diffusion. Each of the four profiles consists of a depressed semicircle and a straight line. The shape of the semicircles indicates that multiple semicircles overlapped. This can be attributed to the interfacial resistance consisting of SEI and charge transfer resistances [47,48]. As cycles increased, the size of the semicircles became larger, indicating that the interfacial resistance increased. This can be related to the accumulation of SEI layers on the active material surface by the repeated cycling. It should be noted that the interfacial resistance values (intermediate frequency intercept) of the Si-CoSi_2_-C electrode were much lower than those of the Si-C electrodes at each cycle. This may be associated with the increased electrical (electronic) conductivity at the electrode surface from the presence of CoSi_2_.

## 4. Conclusions

We demonstrated the enhanced electrochemical properties of a porous Si-CoSi_2_-C composite electrode. The electrode was prepared by a two-step HEMM and cost-effective chemical etching process. The starting materials of Si, Mg_2_Si, and Co_3_O_4_ were converted to Si, CoSi_2_, and MgO phases during the first milling. Super P was incorporated into the composite with the second milling. Finally, the MgO was removed with HCl etching, creating internal pores. The material conversion and synthesis were investigated with XRD, XPS, FE-SEM, TEM, and EDS analyses. BET analysis results exhibited a highly increased specific area and pore volume, proving the presence of internal pores. The composite electrode showed improved cycle and rate performance compared to a reference Si-C composite electrode. These results can be attributed to the CoSi_2_ and the porous composite structure. The highly conductive CoSi_2_ phase improves the electrical connection of the Si to the carbon matrix and current collectors; the CoSi_2_ and structural pores buffer the large Si volume changes during cycling; and the porous structure aids fast Li-ion transport through the electrolyte to the electrode surface. The proposed synthesis strategy can provide a facile and cost-effective method to produce Si-based anodes for commercial LIBs.

## Data Availability

Data available on request.

## Supplementary Materials

The following are available online at https://www.mdpi.com/article/10.3390/ma14185397/s1: Figure S1: Enlarged XRD patterns of the pristine mixture; Figure S2: Raman spectrum of the synthesized composite title; Figure S3: XPS core-level spectra with increasing sputtering time; Figure S4: Enlarged DCPs of the porous Si-CoSi_2_-C composite electrode; Figure S5: Cyclic voltammograms of the composite electrode; Figure S6: FE-SEM images of the cycled electrodes; Figure S7: Enlarged Nyquist plots of the Si-CoSi_2_-C and Si-C composite electrodes.
