# Supplementary material for: Scalable Synthesis and Electrochemical Properties of Porous Si-CoSi2-C Composites as an Anode for Li-ion Batteries
Source: Materials (Basel). 2021 Sep 18;14(18):5397. doi: 10.3390/ma14185397 (PMC8469060; doi:10.3390/ma14185397)
Supplement: Supplementary file 1 [file materials-14-05397-s001.zip › materials-1347250-supplementary.pdf]

Supplementary Materials

Scalable synthesis and electrochemical properties of porous Si-$CoSi_2$-C composites as an anode for Li-ion batteries

Hyungeun Seo [1], Hae-Ri Yang [1], Youngmo Yang [1], Kyungbae Kim [1], Sung Hyon Kim [2], Hyunseung Lee [3], and Jae-Hun Kim [1,*]

[1] School of Materials Science and Engineering, Kookmin University, Seoul 02707, Republic of Korea

[2] Department of Fashion Design, Kookmin University, Seoul 02707, Republic of Korea

[3] Department of Fashion Industry, Incheon National University, Incheon 22012, Republic of Korea

* Correspondence: jaehunkim@kookmin.ac.kr

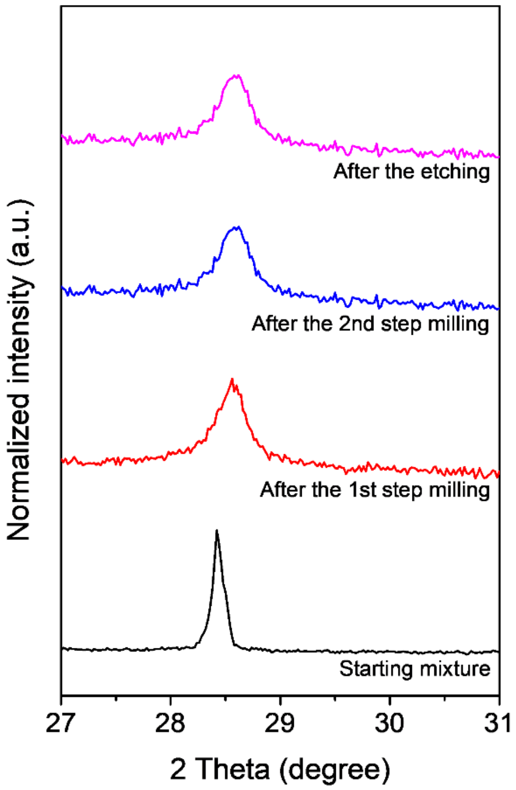

Figure S1. XRD patterns of the pristine mixture and the samples after each synthetic step between 27 and 31 degrees.

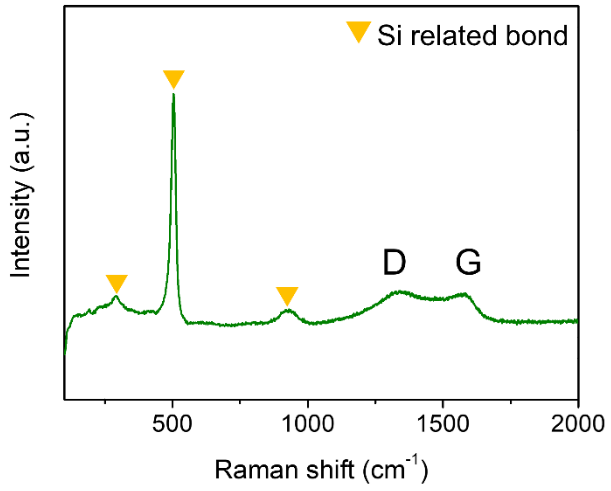

Figure S2. Raman spectrum of the synthesized composite.

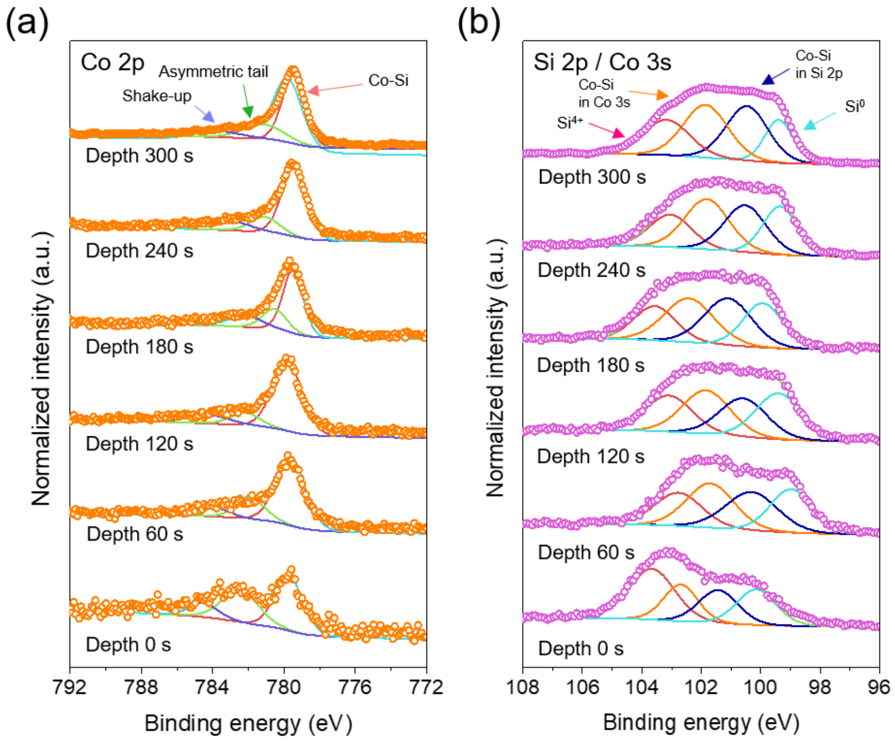

Figure S3. XPS core-level spectra with increasing sputtering time: (a) Co 2p and (b) Si 2p/Co 3s.

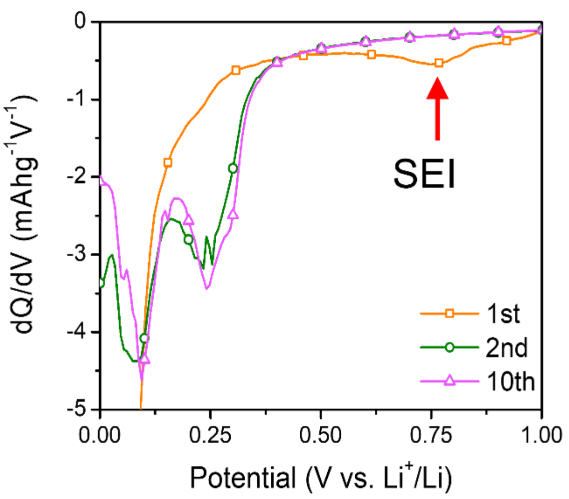

Figure S4. Enlarged DCPs of the porous Si-$CoSi_2$-C composite electrode.

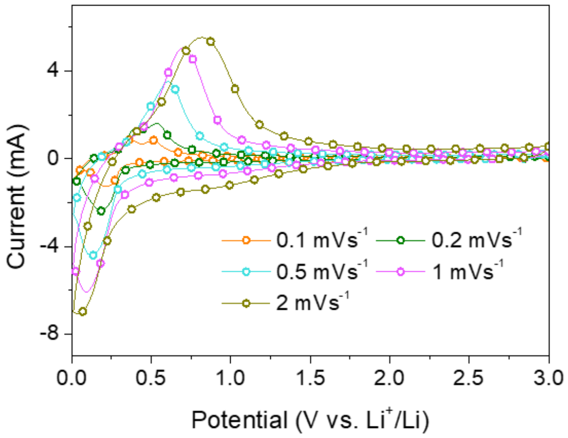

Figure S5. Cyclic voltammograms of the Si-$CoSi_2$-C composite electrode at various scan rates.

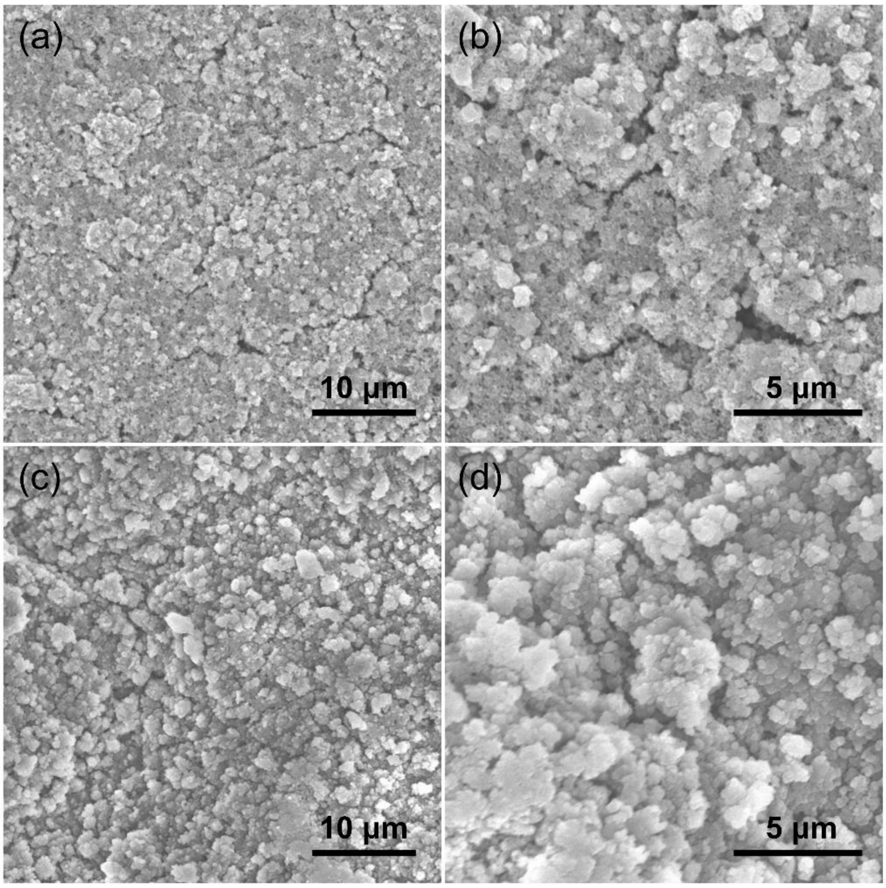

Figure S6. FE-SEM images of the porous Si-$CoSi_2$-C composite electrodes (a, b) before cycling and (c, d) after 100 cycles.

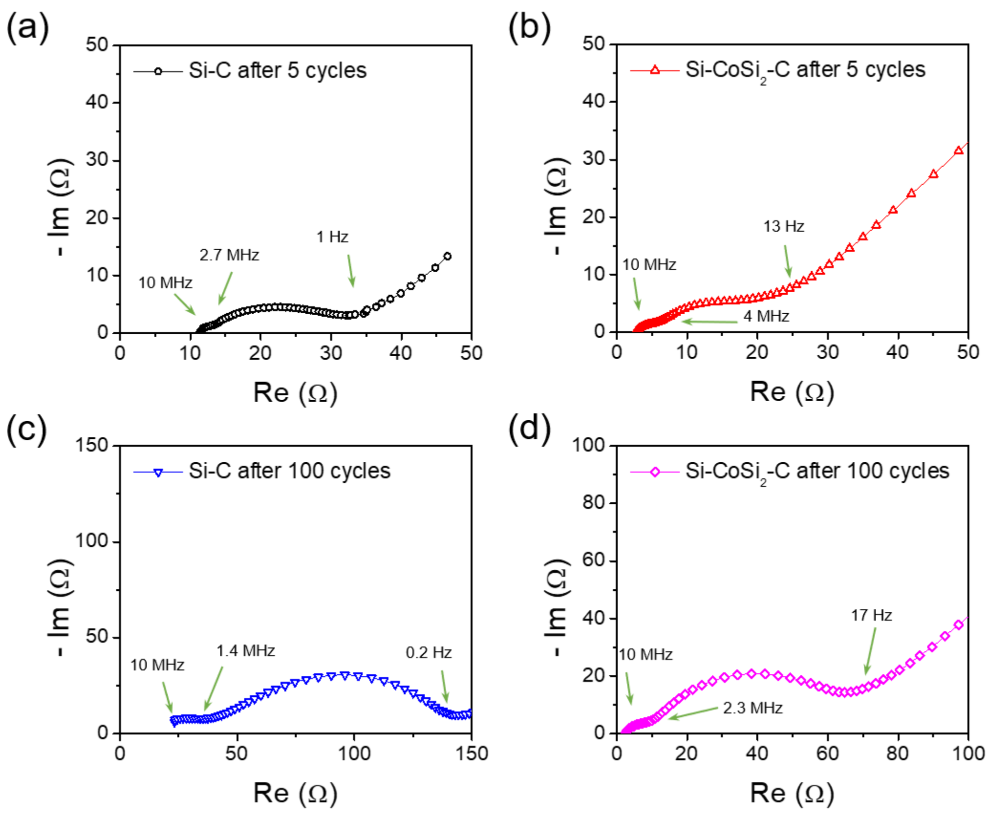

Figure S7. Enlarged Nyquist plots of the (a, c) Si-C and (b, d) Si-$CoSi_2$-C composite electrodes after 5 and 100 cycles.
